# Cochaperones enable Hsp70 to use ATP energy to stabilize native proteins out of the folding equilibrium

**DOI:** 10.1038/s41598-018-31641-w

**Published:** 2018-09-04

**Authors:** Huafeng Xu

**Affiliations:** Unaffiliated, Forest Hills, NY 11375 USA

## Abstract

The heat shock protein 70 (Hsp70) chaperones, vital to the proper folding of proteins inside cells, consume ATP and require cochaperones in assisting protein folding. It is unclear whether Hsp70 can utilize the free energy from ATP hydrolysis to fold a protein into a native state that is thermodynamically unstable in the chaperone-free equilibrium. Here I present a model of Hsp70-mediated protein folding, which predicts that Hsp70, as a result of differential stimulation of ATP hydrolysis by its Hsp40 cochaperone, dissociates faster from a substrate in fold-competent conformations than from one in misfolding-prone conformations, thus elevating the native concentration above and suppressing the misfolded concentration below their respective equilibrium values. Previous models would not make or imply these predictions, which are experimentally testable. My model quantitatively reproduces experimental refolding kinetics, predicts how modulations of the Hsp70/Hsp40 chaperone system affect protein folding, and suggests new approaches to regulating cellular protein quality.

## Introduction

The discovery of chaperones and their roles in assisting protein folding amended the long-held view that proteins spontaneously fold into their native structures^1–3^. Large, multi-domain proteins may take many hours to fold, or fail to fold properly altogether on their own^2,4^. ATP-consuming chaperones—including Hsp70s—provide critical assistance in the *in vivo* folding and the biological functions of broad sets of substrate proteins^3^. Extensive experimental studies have firmly established that within the same length of time, more denatured substrate proteins refold in the presence of the Hsp70 chaperones than in their absence^5,6^. Despite tremendous progress in the mechanistic studies of the Hsp70 chaperones^2,7,8^, including the development of theoretical models^9–12^, it remains unclear why ATP consumption is indispensable to these chaperones; many enzymes catalyze chemical reactions without consuming free energy. Recently it was demonstrated that chaperones such as GroEL and Hsp70 depend on continuous ATP hydrolysis to maintain a protein in a native state that is thermodynamically unstable^13^, but it is unknown how Hsp70 can utilize the ATP free energy to alter the folding equilibrium. In addition, Hsp70s require Hsp40—also known as J proteins^14^—cochaperones in assisting protein folding. It is yet unexplained why cochaperones are absolutely necessary.

The Hsp70 chaperones, such as the bacterial DnaK, consist of an N-terminal nucleotide binding domain (NBD) and a C-terminal substrate binding domain (SBD). The Hsp70 SBD adopts an open conformation when its NBD is ATP-bound (I call the Hsp70 to be in the ATP-state), which allows the substrate to bind and unbind at high rates, whereas when the NBD is ADP-bound (ADP-state), the SBD changes to a closed conformation, rendering both binding and unbinding orders-of-magnitude slower^6,15,16^. Hsp70s have low basal ATP hydrolysis activities^17^. The Hsp40 cochaperones, such as the bacterial DnaJ, can drastically stimulate the ATPase activity of Hsp70 using their N-terminal J domain (JD)^18,19^, shared by all Hsp40s (hence the name J proteins)^14^. Hsp40s also have a C-terminal domain (CTD) that can bind to denatured proteins^20,21^. Both Hsp70 and Hsp40 recognize exposed hydrophobic sites^7,22,23^. As a result, they can distinguish different protein conformations using the corresponding difference in the exposed hydrophobic sites. For example, Hsp70 has been shown to bind to both unfolded and partially folded, near native protein structures, but not to native structures^24,25^. Hsp40 and Hsp70 may simultaneously bind to different segments of the same substrate molecule, and the consequent spatial proximity then facilitates the J domain binding to Hsp70 and accelerating its ATP hydrolysis^26–28^. Following ATP hydrolysis, the chaperone returns from the ADP-state to the ATP-state through nucleotide exchange, which is often catalyzed by nucleotide exchange factors (NEF) such as the bacterial GrpE^29,30^.

It is unclear whether Hsp70 can use the free energy from ATP hydrolysis to drive its substrate protein toward the native state, *N*, and away from the misfolded state, *M*, such that *f*_*N*_*/f*_*M*_ > *f*_*N,eq*_*/f*_*M,eq*_, where *f*_*S*_ is the fraction of the substrate in state *S* at the steady state of Hsp70-mediated folding, and *f*_*S,eq*_ is the corresponding fraction at the folding equilibrium in the absence of the chaperone. Previous models^10,31^ mostly considered the chaperone as an unfoldase/holdase—which need not consume free energy—that pulls the substrate out of the misfolded state and holds it in an unfolded state. It was proposed that the free energy from ATP hydrolysis was used to achieve ultra-affinity in substrate binding^9,32^. As an unfoldase/holdase, Hsp70 would also pull the substrate out of the native state into the unfolded state; unless Hsp70 has a higher affinity for the native substrate than for the misfolded substrate, which contradicts experimental observations, these models would predict *f*_*N*_*/f*_*M*_ ≤ *f*_*N,eq*_*/f*_*M,eq*_.

Here I propose a model of Hsp70-mediated protein folding, in which Hsp70 and Hsp40 together constitute a molecular machine that uses the free energy from ATP hydrolysis to actively drive a protein toward its native state, so that *f*_*N*_*/f*_*M*_ > *f*_*N,eq*_*/f*_*M,eq*_. It suggests that without Hsp40, Hsp70 alone cannot change the ratio *f*_*N*_*/f*_*M*_ from the equilibrium value *f*_*N,eq*_*/f*_*M,eq*_. My model thus answers the question why Hsp70 requires both the Hsp40 cochaperones and ATP consumption in assisting protein folding. My model explains the puzzling non-monotonic dependency of folding efficiency on the chaperone and cochaperone concentrations. It makes quantitative predictions on how protein folding is affected by modulations of the chaperone environment, including changes in the ATPase activity or the nucleotide exchange rate of Hsp70. These predictions may be readily tested by experiments, and inform rational approaches to manipulating chaperone-mediated protein folding.

## Results

**Figure 1:**
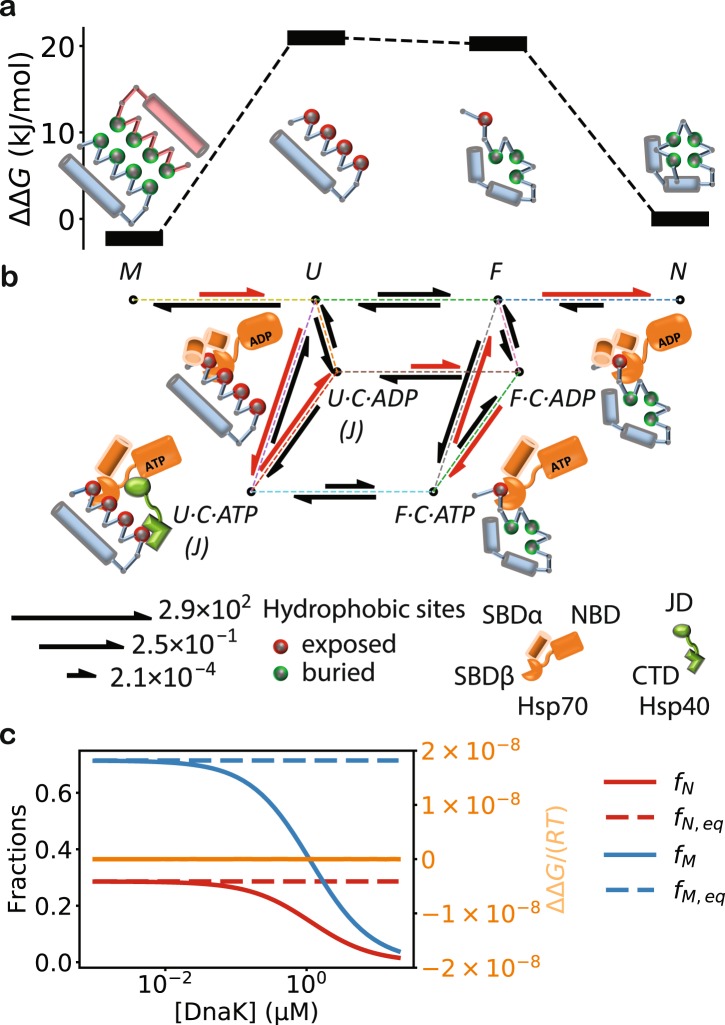
My model of Hsp70/Hsp40/NEF-mediated protein folding. (**a**) Conformational states in protein folding, and their relative free energies in the absence of chaperones. I assume that there are four conformational states: *M*isfolded, *U*nfolded and misfolding-prone, *F*old-competent, and *N*ative. The pink and blue chains in the *M* state may correspond to different molecules (reversibly aggregated) or to different domains in the same molecule. The high free energy barriers associated with the intermediate states *U* and *F* slow down the refolding of misfolded proteins. The exemplary free energies are derived from the kinetic parameters fit to the refolding experiments of luciferase at 25 °C (Table 2). (**b**) The transitions between the microscopic states in the chaperone-mediated folding pathway. *S*·*C*·*X* represents the complex between the substrate in the conformational state *S* (=*U*, *F*) and the Hsp70 chaperone (denoted as *C*) bound to nucleotide X (=*ATP*, *ADP*). The transitions between *S* and *S*·*C*·*X* correspond to the chaperone binding to and unbinding from the substrate. The transition of *S*·*C*·*ATP* to *S*·*C*·*ADP* corresponds to ATP hydrolysis, and its reverse, nucleotide exchange. Hsp70 binding stabilizes the substrate in the intermediate states, thus catalyzing the folding reaction. Hsp40 (*J*) can form a ternary complex with the substrate and Hsp70—thus stimulating ATP hydrolysis—if the substrate is in the *U* state, but not if the substrate is in the *F* state. Differential ATP hydrolysis by Hsp70 bound to the substrate in the *U* and *F* states drives the refolding through the pathway highlighted in red. The lengths of the reaction arrows are linear with respect to the logarithms of the exemplary rate constants (in 1/s) for the DnaK/DnaJ/GrpE-mediated refolding of luciferase at 25 °C (Tables 1 and 2). (**c**) Without cochaperones, Hsp70 cannot alter the balance between folding and misfolding. DnaK binding to the intermediate states decreases both the native (red) and misfolded (blue) populations, but the ratio between the two remains unchanged from its equilibrium value: ΔΔ*G* = 0 (orange, right y-axis). Here, I have taken ${k}_{a,C\cdot ATP}^{(U)}/{k}_{a,C\cdot ATP}^{(F)}={k}_{a,C\cdot ADP}^{(U)}/{k}_{a,C\cdot ADP}^{(F)}=100$ to show that differential binding of Hsp70 to the substrate in different conformational states does not alter the folding/misfolding balance.

My model is based on two assumptions supported by experimental observations. The first assumption is that a substrate protein can adopt two additional conformational states besides the misfolded, *M*, and the native, *N*, states: the unfolded and misfolding-prone state, *U*, and the fold-competent state, *F*. A protein in the *F* state is unfolded but poised to fold into the native state (Fig. 1a,b). Such intermediate states of folding have been observed experimentally^4^. Conformational transitions can occur between *M* and *U*, between *U* and *F*, and between *F* and *N* (Fig. 1b). The second assumption is that Hsp40’s affinity for a substrate protein is higher if the substrate is in the misfolding-prone conformation than if it is in the fold-competent conformation. Experimental observations suggest that the misfolding-prone conformation is less compact and exposes more hydrophobic sites—thus providing more accessible sites for Hsp40 binding—than the fold-competent conformation^4^. Consequently, a substrate in the *U* state is more likely to be Hsp40-bound than one in the *F* state.

**Table 1:** The experimental kinetic parameters for the DnaK/DnaJ/GrpE chaperone system.

Reaction	Parameter	Rate	Reference
$S+C\cdot ATP\rightleftharpoons S\cdot C\cdot ATP$	${k}_{a,C\cdot ATP}^{(0)}$	1.28 × 10^6^ M^−1^·s^−1^	^6^
	${k}_{d,C\cdot ATP}$	2.31 s^−1^	^6^
$S+C\cdot ADP\rightleftharpoons S\cdot C\cdot ADP$	${k}_{a,C\cdot ADP}^{(0)}$	1.17 × 10^4^ M^−1^·s^−1^	^6^
	${k}_{d,C\cdot ADP}$	9.1 × 10^−4^ s^−1^	^6^
$J+C\cdot ATP\rightleftharpoons J\cdot C\cdot ATP$	${k}_{a,J\cdot C}$	4.88 × 10^4^ M^−1^·s^−1^	^17a^
	${k}_{d,J\cdot C}$	1.6 × 10^−3^ s^−1^	^51^
$S\cdot C\cdot ATP\to S\cdot C\cdot ADP+Pi$	${k}_{h}^{(0)}$	0.01 s^−1^	^17b^
$J\cdot S\cdot C\cdot ATP\to J\cdot S\cdot C\cdot ADP+Pi$	${k}_{h}^{(J)}$	1.8 s^−1 c^	^17^
$C\cdot ADP\to C+ADP$	${k}_{d,ADP}$	0.006 s^−1^	^52^
$E+C\cdot ADP\rightleftharpoons E\cdot C\cdot ADP$	${k}_{a,E\cdot C}$	7.85 × 10^6^ M^−1^·s^−1^	^29d^
	${k}_{d,E\cdot C}$	30 s^−1^	^29^
${C}_{{\rm{closed}}}\cdot ATP\to {C}_{{\rm{open}}}\cdot ATP$	${k}_{C}^{(0)}$	1.1 × 10^23^ s^−1^	^50e^
	${E}_{a}/R$	17320 *K*	^50^

All the parameters are determined at the temperature of 25 °C, except for the rate of conformational change of DnaK from closed to open conformations, which was measured at 15 °C, 25 °C, and 35 °C, yielding the parameters ${k}_{C}^{(0)}$ and ${E}_{a}$ from a fitting of the Arrhenius equation to the measured rates at different temperatures.

^a^The association rate constant of DnaJ binding to DnaK was determined from the DnaJ concentration dependence of the DnaK ATP hydrolysis rates, by fitting the hydrolysis rate $${k}_{h}([J])={K}_{h}{(1+{K}_{h})}^{-1}{k}_{h}^{(J,w/o\,{\rm{S}})}+{(1+{K}_{h})}^{-1}{k}_{h}^{(0,w/o\,S)}$$ where $${K}_{h}\equiv {k}_{a,J\cdot C}\cdot [J]/({k}_{d,J\cdot C}+{k}_{h}^{(J,w/o\,{\rm{S}})})$$, to the experimental data with respect to $${k}_{a,J\cdot C}$$, $${k}_{h}^{(J,w/o\,S)}$$, and $${k}_{h}^{(0,w/o\,{\rm{S}})}$$, yielding the $${k}_{a,J\cdot C}$$ value in the table, $${k}_{h}^{(J,\,w/o\,{\rm{S}})}=$$$$0.76\,{s}^{-1}$$, and $${k}_{h}^{(0,\,w/o\,{\rm{S}})}=6\times {10}^{-4}{s}^{-1}$$. I note that the hydrolysis rates are measured without a protein substrate (indicated by the superscript w/o S), which can further accelerate the hydrolysis. Surface plasmon resonance measurement of DnaJ binding to DnaK yielded $${k}_{a,J\cdot C}=2.3\times {10}^{4}{M}^{-1}\cdot {s}^{-1}$$^51^, in good agreement with our fit.

^b^Substrate-free DnaK has a basal ATP hydrolysis rate of 0.001 s^−1^, but the substrate can further accelerate ATP hydrolysis by up to 9-fold^17,53^. I take the ATP hydrolysis rate of substrate-bound DnaK to be 10-fold higher than the basal rate. The predictions of my model are insensitive to this parameter.

^c^This is the experimental rate for the temperature *T* = 25 °C. For the higher temperature *T* = 30 °C, I used an arbitrary but reasonable 5.5-fold higher value of 10 s^−1^ because I could not find any reported experimental value for this temperature.

^d^The kinetic rates for GrpE binding to DnaK were not determined, but the steady state ratio in the two-step dissociation reaction, $$C\cdot ADP+$$$$E\rightleftharpoons E\cdot C\cdot ADP\rightleftharpoons E\cdot C+ADP$$, $${K\text{'}}_{D}=({k}_{d,E\cdot C}+{k}_{d,ADP})/{k}_{a,E\cdot C}=20\,\mu M$$ was determined. I chose an arbitrary diffusion limited association rate for GrpE binding to DnaK in our calculations.

^e^I note that ${k}_{C}^{(0)}$ and ${E}_{a}$ appear too large to be physically meaningful; they should instead be taken simply as numerical parameters that yield an excellent fit of the Arrhenius equation to the experimental data.

The key idea of my model is a mechanism by which Hsp70 actively drives a substrate toward the native state and away from the misfolded state. Based on the above assumptions, an Hsp70 molecule bound to a substrate molecule in the *U* state will on average have substantially higher ATP hydrolysis rate—because of the higher probability of *cis* stimulation by an Hsp40 molecule bound to the same substrate molecule—than if it is bound to a substrate molecule in the *F* state. If the nucleotide exchange rate is between these two hydrolysis rates, an Hsp70 bound to a substrate in the *U* state will be driven toward the ADP-state, where it slowly dissociates from the substrate, while an Hsp70 bound to a substrate in the *F* state will be driven toward the ATP-state, where it rapidly dissociates from the substrate. This enables Hsp70, when bound to substrate molecules, to act like a Maxwell’s demon^33^: it quickly releases the fold-competent molecules so that they can fold, but it retains the misfolding-prone molecules to prevent misfolding. By this mechanism, Hsp70 drives the folding along the reaction path of *M* → *U* → *U* · *C* · *ATP* → *U* · *C · ADP* → *F* · *C* · *ADP* → *F* · *C* · *ATP* → *F* → *N*, where *S* · *C* · *X* represents the complex between a substrate in conformation *S* and the chaperone *C* bound to nucleotide *X* = *ATP*, *ADP* (Fig. 1b). One ATP molecule is consumed in this reaction path and the free energy is used to compel the substrate into the native state.

My model predicts that Hsp70 need Hsp40 in order to alter the folding equilibrium. For Hsp70 to drive substrate folding toward the native state, the above mechanism does not require that more Hsp70 molecules bind to a substrate in the *U* state than to a substrate in the *F* state, which is true and reflected in previous models; instead, it requires that an individual Hsp70 molecule, when bound to a substrate, dissociates slower if the substrate is in the *U* state than if it is in the *F* state. Hsp40 thus plays a critical role because a substrate-bound Hsp70 distinguishes, probabilistically, between the *U* and *F* states of the substrate by sensing whether an Hsp40 is also bound to the same substrate. Defining the excess free energy of folding as

$${\rm{\Delta }}{\rm{\Delta }}G\equiv RT(\mathrm{ln}({f}_{N}\,/{f}_{M})-\,\mathrm{ln}({f}_{N,eq}/{f}_{M,eq})),$$

where *R* is the gas constant and *T* the temperature, it can be shown algebraically (see *Methods*) and numerically (Fig. 1c) that without cochaperones, ΔΔ*G* = 0. This prediction is consistent with the results from the single-molecule experiment of DnaK-mediated refolding^34^, where DnaK alone in the presence of ATP was unable to alter the ratio of the misfolded and folded fractions.

In order to simplify calculations using my model, I assume that a protein’s hydrophobic binding sites for Hsp40 and Hsp70—which can be exposed in the *U* and *F* states—become entirely buried upon folding (*F* → *N*) and misfolding (*U* → *M*), thus a protein in the *M* and *N* states has zero exposed hydrophobic binding sites, and it does not bind to either Hsp70 or Hsp40 (Fig. 1a). The hydrophobic burial in the misfolded state may be due to intramolecular contacts between incorrectly folded domains within a monomeric protein, or due to intermolecular contacts between different protein molecules as a result of oligomerization or mild, reversible aggregation. A protein may also aggregate irreversibly, and will not refold even in the presence of the chaperone system^5^. A protein in such an irreversibly aggregated state may have a prohibitive kinetic barrier to return to the *M* or *U* states. It is thus unamenable to Hsp70-assisted refolding and not included in my model. The quantitative details of my model are given in *Methods*.

**Figure 2:**
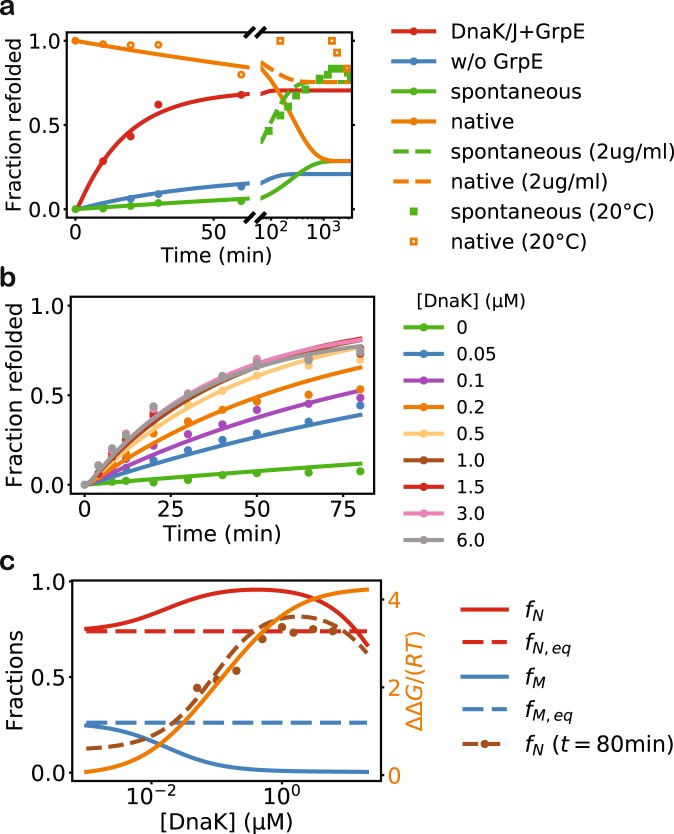
My model is in good agreement with previous experimental studies of DnaK/DnaJ/GrpE-mediated refolding. (**a**) The refolding of denatured luciferase under various conditions. The predictions of my model are shown in lines, whereas the experimental data are shown as filled circles^5^. The dashed lines show the spontaneous refolding and denaturation at the much lower substrate concentration of 0.032 μM (2 μg/ml), as in the corresponding experiments (empty circles and squares)^4^. The experiments of spontaneous refolding and denaturation were performed at 20 °C, lower than the temperature of 25 °C at which most of the kinetic parameters were obtained. In modeling the refolding, I assume that initially all the protein is in the misfolded state, *i*.*e*., *f*_*M*_ (*t* = 0) = 1; in modeling the denaturation, I assume that initially all the protein is in the native state, *i*.*e*., *f*_*N*_ (*t* = 0) = 1. (**b**) The refolding of a luciferase mutant, LucDHis6^31^, in the presence of DnaK at various concentrations. (**c**) The native fraction (*f*_*N*_ = [*N*]/[*S*], red) and the misfolded fraction (*f*_*M*_ = [*M*]/[*S*], blue) of LucDHis6 at the steady state of DnaK-mediated refolding at various DnaK concentrations. The corresponding fractions in the chaperone-free folding equilibrium, *f*_*N,eq*_ and *f*_*M,eq*_, are shown as dashed lines. The unitless excess free energy ΔΔ*G*/(*RT*) is shown in orange (right y-axis). The fractions after 80 min of refolding, starting from misfolded LucDHis6, are shown in brown. My model is in good agreement with the experimental data (filled circles), and it suggests that the refolding is still incomplete even after 80 min. The fitting parameters in my model are given in Table 2, and the conditions of the experiments considered in this paper are summarized in Table 3.

**Figure 3:**
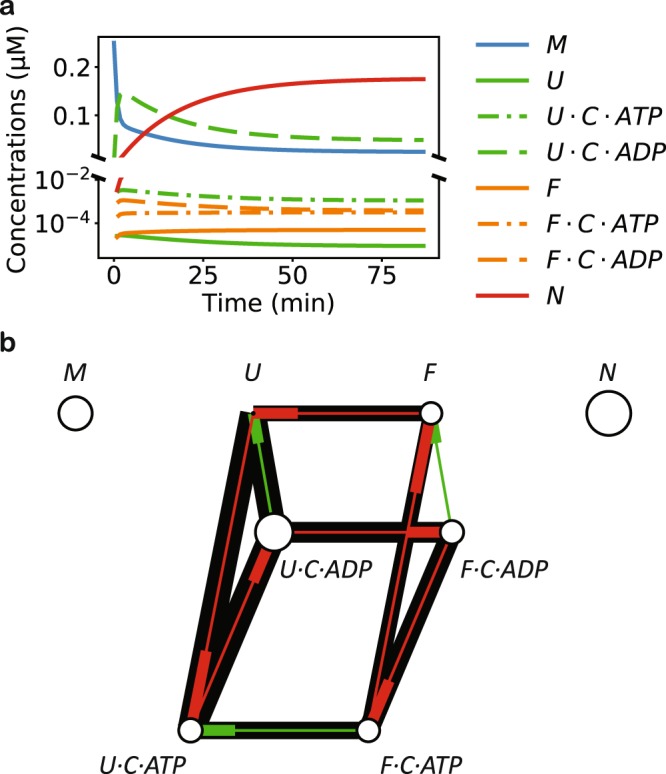
The mechanism by which the Hsp70 chaperone system accelerates refolding and maintains the folding out of equilibrium. (**a**) The model prediction of the concentrations of different molecular species in DnaK/DnaJ/GrpE-mediated refolding of denatured luciferase. The substrate is primarily held by ADP-bound chaperone in the *U* state before slowly moving into the native state. (**b**) The reactive flux at the steady state of LucDHis6 maintained by the DnaK/DnaJ/GrpE chaperone system. The thickness of each line is linear with respect to the logarithm of the absolute value of the reactive flux, and the thicker end of the thin, center line indicates the destination of the flux. The size of the circle at each node is linear with respect to the logarithm of the steady state fraction of the corresponding molecular species. The ATP-driven reaction cycle is highlighted in red.

I applied my model to the analysis of DnaK/DnaJ/GrpE-mediated refolding of luciferase^5^. Most of the relevant kinetic parameters at 25 °C for this bacterial Hsp70 system have been carefully determined experimentally^35^ (Table 1). My model quantitatively reproduces the experimentally observed refolding kinetics under various conditions, capturing the slow spontaneous refolding and denaturation of luciferase, the acceleration of refolding with chaperone assistance, and the necessity of GrpE (Fig. 2a). The intermediate conformations *U* and *F* in my model in the case of luciferase may correspond to the experimentally identified intermediate conformations *I*_2_ and *I*_1_ of luciferase^4^: the free energy difference between *N* and *F* at 25 °C, according to the fitted parameters, is 20 kJ/mol, close to the experimental value of 15 kJ/mol between *N* and *I*_1_, measured at 10 °C. Consistent with previous experimental observations^31^, my model suggests that the Hsp70-mediated refolding proceeds in two steps: (1) rapid unfolding of the misfolded substrate, stabilized by the ADP-bound DnaK, followed by (2) slow conversion to the native state (Fig. 3a).

**Table 2:** The model parameters fit to the refolding experiments.

Reaction	Parameter	Luciferase		LucDHis6
		25 °C	30 °C	22 °C
$M\rightleftharpoons U$	${k}_{M\to U}\,({s}^{-1})$ ^a^	0.02	0.02	0.02
	${k}_{U\to M}({M}^{-1}\cdot {s}^{-1})$	9.4 × 10^8^	3.4 × 10^9^	6.4 × 10^7^
$U\rightleftharpoons F$	${k}_{U\to F}\,({s}^{-1})$	0.23	0.88	0.26
	${k}_{F\to U}\,({s}^{-1})$	0.18	0.13	0.042
$F\rightleftharpoons N$	${k}_{F\to N}\,({s}^{-1})$ ^b^	5	5	5
	${k}_{N\to F}({10}^{-3}\cdot {{\rm{s}}}^{-1})$	1.4	20	1.1
$S+C\cdot X\rightleftharpoons S\cdot C\cdot X$	${n}_{C}^{(U)}$	9.1	6.2	1.9
	${n}_{C}^{(U)}/{n}_{C}^{(F)}$ ^c^	20	20	20
$S+J\rightleftharpoons S\cdot J$	${K}_{A,J}^{(U)}({10}^{6}\cdot {M}^{-1})$	13	22	54
$(J+C)\cdot S\rightleftharpoons (J\cdot C)\cdot S$	${[J]}_{{\rm{eff}}}$ (M)^d^	5 × 10^−3^	5 × 10^−3^	5 × 10^−3^

^a^The predictions of my model depend on the equilibrium constant ${K}_{M\rightleftharpoons U}={k}_{M\to U}/{k}_{U\to M}$, but are degenerate with respect to the individual rates. I thus fix an arbitrary but plausible value for ${k}_{M\to U}$ and fit ${k}_{U\to M}$ to the experimental refolding data.

^b^For the similar reason as above, I fix ${k}_{F\to N}$ and fit ${k}_{N\to F}$ to the refolding data.

^c^There should be more accessible DnaK binding sites in the *U* state than in the *F* state. Here, I arbitrarily set the ratio between the two. Although the values of the other fitting parameters will change accordingly, the quality of the fit and the predictions of my model are insensitive to this ratio (at least for values between 1 and 100).

^d^I assume that the effective distance, *L*, between the Hsp70 molecule and the J domain of the Hsp40 molecule bound to the same substrate molecule is *L* = 4.3 nm, the effective concentration is then ${[J]}_{{\rm{eff}}}={N}_{A}^{-1}/({L}^{3}4\pi /3)=5\times {10}^{-3}M$. The quality of the fit and the predictions of my model are insensitive to this parameter.

Next, I used my model to analyze how the refolding kinetics and yield change with respect to the DnaK concentration, which have been experimentally studied for LucDHis6, a variant of luciferase^31^. The refolding data fits well with the kinetic equation of a first-order reaction, $N(t)=N(\infty )(1-\exp (-kt))$, and the rate constant *k* is approximately unchanged across different DnaK concentrations^8,36^. DnaK thus affects the refolding kinetics mostly by altering the refolding yield $N(\infty )$. My model captures the refolding kinetics and the refolding yield of LucDHis6 in the experimental range of DnaK concentrations (Fig. 2c); although the experiments were performed at the temperature of 22 °C, the predictions of my model using the kinetic parameters derived for 25 °C are nevertheless in quantitative agreement with the experimental data. At the steady state, the reactive flux along the ATP-driven cycle *U* → *U · C · ATP* → *U · C · ADP* → *F · C · ADP* → *F · C · ATP* → *F* ( → *U*) (Fig. 3b) keeps the protein folding out of equilibrium, elevating the native population above and suppressing the misfolded population below their respective equilibrium values (Fig. 2c). Notably, the refolding yield peaks around [DnaK] = 1 µM, and it decreases at higher DnaK concentrations. This non-monotonic dependence on the DnaK concentration was also reported for the wildtype luciferase while this work was under review^36^. According to my model, the excess free energy at the steady state always increases with increasing DnaK concentrations, but the native population reaches a maximum and then decreases (Fig. 2c), because at high DnaK concentrations, the substrate is trapped in the DnaK-bound state and thus prevented from folding into the native state.

**Table 3:** The conditions of the refolding experiments (the corresponding references are in superscripts).

	A^4^	B^5^	C^17^	D^29^	E^31^
[DnaK] (µM)	0	1.25	0.8	0.8	0 to 6
[DnaJ] (µM)	0	0.25	0 to 0.8	0.16	0.3
[GrpE] (µM)	0	1.25	0.4	0 to 4	0.8
[S] (µM)	0.0324	0.25	0.08	0.08	3
[ATP] (mM)	0	1	5	N/A	5
[ADP] (mM)	0	0	0	N/A	0
T (°C)	20, 30	25	30	30	22

A: Spontaneous refolding of guanidinium chloride (GdmCl)-denatured luciferase. B–D: DnaK/DnaJ/GrpE-mediated refolding of GdmCl-denatured luciferase. E: DnaK/DnaJ/GrpE-mediated refolding of LucDHis6 denatured by 4 freeze-thaw cycles. LucDHis6 is an engineered luciferase variant where the last 62 COOH-terminal residues are replaced by SKLSYEQDGLHAGSPAALEHHHHHH-COOH.

**Figure 4:**
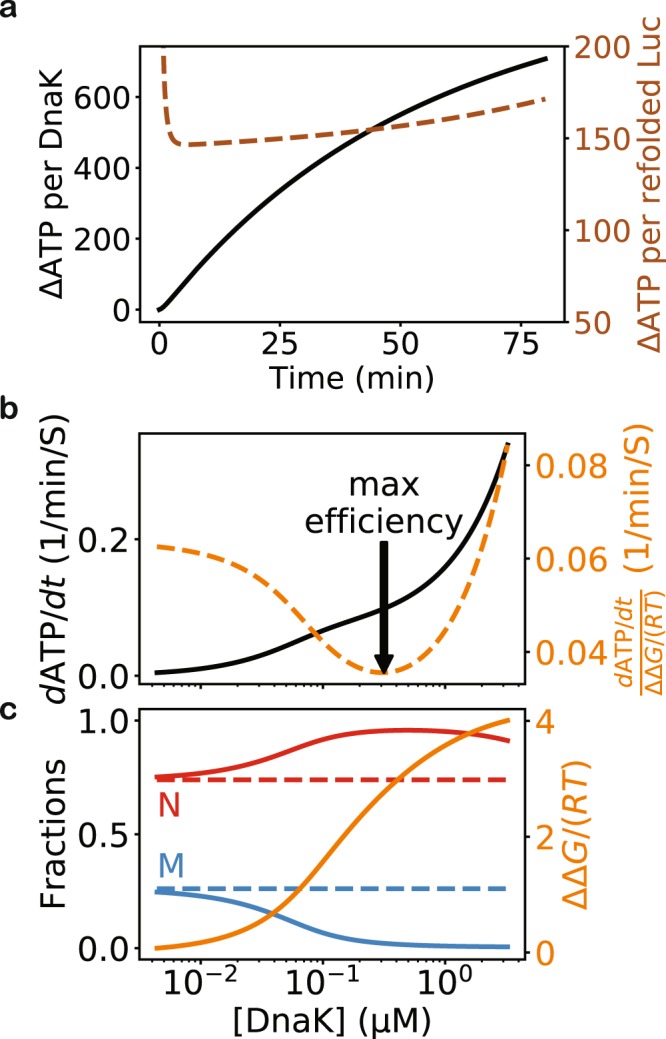
Free energy consumption in DnaK/DnaJ/GrpE-mediated folding of LucDHis6. (**a**) ATP consumption in the course of refolding of denatured LucDHis6. The instantaneous rate of ATP consumption is given by $d{\rm{ATP}}/dt=\sum _{S\in \{U,F\}}{k}_{h}^{(S)}([J])\cdot [S\cdot C\cdot ATP]$, and the number of hydrolyzed ATP per molecule of refolded substrate can be estimated by dividing the cumulative consumption, ${\rm{\Delta }}\mathrm{ATP}={\int }_{0}^{\tau }d{\rm{ATP}}$, by the number of refolded substrate molecules after time *τ*. Here, the DnaK concentration is 0.5 μM, and the other kinetic parameters are given in Tables 1 and 2. The black curve shows the number of ATP molecules hydrolyzed per DnaK molecule after the given time. The brown curve shows the number of ATP molecules consumed per one molecule of refolded LucDHis6 (right y-axis) up to the given time, which increases to infinity at the steady state because no additional LucDHis6 is refolded, yet ATP hydrolysis continues. (**b**,**c**) ATP hydrolysis at the steady state. The ATP consumption rate per substrate, at various DnaK concentrations, is shown as the black curve in panel b, and the corresponding native (red) and misfolded (blue) fractions at the steady state are shown as solid lines in panel c. The native fraction is above and the misfolded fraction is below their respective equilibrium values (dashed flat lines). The excess free energy ΔΔ*G*/(*RT*) is shown in orange (right y-axis) in panel c. I can measure the chaperones’ free energy efficiency in maintaining the non-equilibrium by the ratio of the ATP consumption rate to the excess free energy at the steady state (orange, right y-axis, in panel b). The arrow indicates the DnaK concentration at which the chaperones utilize the least amount of ATP per unit of excess free energy.

I used my model to estimate the ATP consumption in the DnaK/DnaJ/GrpE-mediated folding, which has been experimentally measured for LucDHis6^31^ (Fig. 4). My model estimates that, in the initial minutes of refolding, approximately 150 ATP molecules are consumed to refold one LucDHis6 (Fig. 4a), which is reasonably close to the experimental result of ~50 ATP molecules consumed per refolded LucDHis6 when the stoichiometry of DnaK:LucDHis6 is 1:1, significantly higher than the experimental number of ~5 when LucDHis6 is in excess of DnaK, and significantly lower than the estimates of >1000 for many other substrates in other experiments^31,37–39^. The discrepancy between the model and the experimental results may be attributable to the approximations in my model and the inaccuracies in the input kinetic parameters. ATP hydrolysis continues at the steady state and the free energy is utilized to promote the native state and suppress the misfolded state (Fig. 4b,c). As [DnaK] exceeds 1 µM, the ATP consumption rate increases rapidly without commensurate increase in the excess free energy. My analysis thus suggests that DnaK may be most free energy efficient at maintaining protein folding out-of-equilibrium when its concentration is in the sub-micromolar range, a prediction that may be tested experimentally.

**Figure 5:**
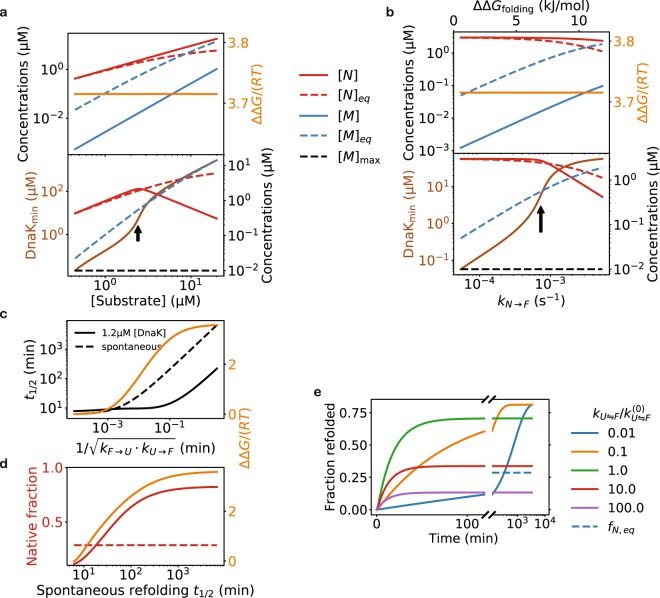
The capacity of the DnaK/DnaJ/GrpE chaperones to prevent reversible aggregation and to elevate native protein concentrations. (**a**) The chaperones can prevent aggregation at increasing substrate concentrations. The rate of aggregation is taken to be proportional to the substrate concentration (see *Methods*). Top: the native and the misfolded steady state concentrations at increasing total LucDHis6 concentrations, with the DnaK concentration fixed at 1.2 μM. Bottom: the DnaK concentration required to maintain the misfolded protein concentration at or below [*M*]_max_ = 0.01 µM, as well as the steady state concentration of the native substrate at that DnaK concentration. (**b**) The chaperones can prevent aggregation at decreasing substrate stability. I vary the protein stability by changing the rate constant of conversion, *k*_*N*→*F*_, from the *N* state to the *F* state; the corresponding change in the folding free energy ΔΔ*G*_folding_ is indicated on the top axis. The native and the misfolded concentrations, as well as the DnaK concentration required to prevent aggregation, are shown as in panel a. (**c**,**d**) Hsp70 is more efficient at folding substrates with slower conversion between the *U* and the *F* states. Here, I take the kinetic parameters of luciferase folding at 25 °C, and simultaneously scale the forward and reverse rates of the reaction $U\rightleftharpoons F$ by the same factor, thus changing the kinetics without affecting the folding equilibrium. The times, *t*_1/2_, for the refolding of the misfolded substrate to reach half of the native fraction at equilibrium (spontaneous refolding) or the steady state (mediated by DnaK/DnaJ/GrpE), as well as the excess free energy (orange, right y-axis), are plotted against the hypothetic rates of conversion in c. The native fractions (red, left y-axis) and the excess free energy (orange, right y-axis) at the steady state are plotted against *t*_1/2_ of spontaneous refolding in d; the equilibrium native fraction is shown as the red dotted line. (**e**) The time courses of Hsp70-mediated refolding of the misfolded substrate at different hypothetical rates of conversion between *U* and *F* (keeping *k*_*U*→*F*_/*k*_*F*→*U*_ constant). Higher steady state native fractions are obtained at the price of longer refolding times.

My model suggests that Hsp70 can keep a protein folded even if it thermodynamically tends to misfold and aggregate (Here I only consider reversible aggregation). The chaperone is thus able to play a critical role in maintaining protein conformations, not just in the folding of nascent chains^40^. Higher DnaK concentrations are required to suppress aggregation at increasing substrate concentrations (Fig. 5a) or at decreasing substrate stabilities (Fig. 5b). This may explain how cells that overexpress DnaK can tolerate higher numbers of mutations in the chaperone’s substrates^41^. Because the excess free energy plateaus at high chaperone concentrations (Fig. 2c), my results imply a limit on the chaperones’ capacity to prevent aggregation, in that there exists a threshold of aggregation tendency (Fig. 5a,b, the black arrows) above which the chaperone can no longer maintain high levels of native concentrations and prevent misfolding/aggregation at the same time.

My model suggests that Hsp70 only drives the folding of proteins with sufficiently slow conversion between *U* and *F* states (Fig. 5c–e), implying that Hsp70 substrates tend to be slow refolding proteins (Fig. 5d). If the conversion between *U* and *F* is too fast, the chaperone diminishes, rather than increases, the native fraction in comparison to the chaperone-free equilibrium. As the conversion slows, the chaperone drives the steady state native fraction higher, but at the price of longer refolding time (Fig. 5e), a trade-off reminiscent of that between speed and specificity in the kinetic proofreading mechanism^42,43^, where the expenditure of free energy (such as from ATP or GTP consumptions) is utilized to increase the specificity of chemical reactions.

**Figure 6:**
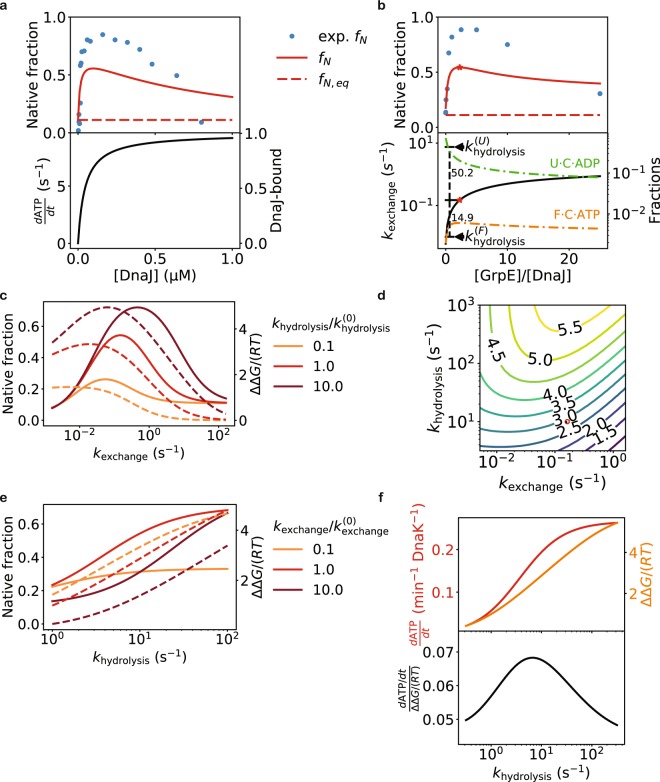
The rates of Hsp40-catalyzed ATP hydrolysis and NEF-catalyzed nucleotide exchange affect the efficiency of Hsp70-mediated folding. (**a**) Refolding of luciferase at various DnaJ concentrations. Top: the steady state native fractions predicted by my model, compared to the experimental data of refolding after 30 min at 30 °C^17^. Bottom: the ATP hydrolysis rate (left y-axis) and the fraction of substrate bound to DnaJ (right y-axis). (**b**) Refolding of luciferase at various GrpE concentrations. Top: the steady state native fractions predicted by my model, compared to the experimental data of refolding after 2 hours at 30 °C^29^. Bottom: the rate of nucleotide exchange at different GrpE concentrations (black line, left y-axis), and the populations of *F · C · ATP* and *U · C · ADP* (right y-axis). The optimal GrpE concentration is indicated by the red stars, and the in-plot numbers show the corresponding ratios of the nucleotide exchange rate to the ATP hydrolysis rates in the *U* and *F* states. In a and b, the rates of GrpE-catalyzed nucleotide exchange and DnaJ-catalyzed ATP hydrolysis are adjusted for the temperature of 30 °C (see *Methods* and Table 1). (**c**) Folding efficiency at different hypothetical rates of nucleotide exchange, for different values of the ATP hydrolysis rate in the *U* state. Native fractions (solid lines, left y-axis) are diminished at both low and high nucleotide exchange rates. At high rates of nucleotide exchange, the excess free energies (dashed lines, right y-axis) approach zero, indicating that Hsp70 can no longer drive protein folding. (**d**) The excess free energy as a function of the nucleotide exchange and the DnaJ-catalyzed ATP-hydrolysis rates. The rates used to model DnaK/DnaJ/GrpE-mediated folding at 30 °C are indicated by the red circle. (**e**) Folding efficiency increases with the DnaJ-catalyzed ATP hydrolysis rate, yielding higher native fractions (solid lines, left y-axis) and larger excessive free energies (dashed lines, right y-axis). (**f**) Higher ATP hydrolysis rate yields larger excess free energy (orange, right y-axis, top), at the price of higher rate of ATP consumption (red, left y-axis, top). The ratio of the two (bottom) changes only slightly.

My model explains the observation that folding is less efficient at both low and high DnaJ concentrations^17^ (Fig. 6a). At low DnaJ concentrations, ATP hydrolysis is slow, and nucleotide exchange drives DnaK toward the ATP-state, in which it dissociates from the substrate rapidly and thus unable to prevent aggregation. At high DnaJ concentrations, a large fraction of the substrate in the *U* state is bound to DnaJ. These DnaJ-bound substrate molecules are trapped in the *U* state, unable to progress toward the *F* state, resulting in diminished folding.

My model also explains the observation that folding decreases at both low and high GrpE concentrations^29^ (Fig. 6b). For the chaperone to effectively assist folding, nucleotide exchange should be much slower than ATP hydrolysis when the chaperone binds to a substrate in the *U* state, but much faster than ATP hydrolysis when it binds to a substrate in the *F* state, so that the chaperone is driven toward the ADP-state in the former case, and toward the ATP-state in the latter case (Fig. 1b). At low GrpE concentrations, nucleotide exchange is slow, leaving DnaK bound to the substrate in the *F* state predominantly in the ADP-state—as reflected by the low population of *F · C · ATP* (Fig. 6b), slowing its dissociation from the substrate and thus preventing the latter from folding to the native state. At high GrpE concentrations, nucleotide exchange is fast, and DnaK is driven into the ATP-state and does not stay bound to the substrate in the *U* state long enough—as reflected by the decreasing population of *U · C · ADP* (Fig. 6b)—to prevent the substrate from aggregation. To maximize substrate folding, higher nucleotide exchange rate should accompany higher stimulated ATP hydrolysis rate (Fig. 6c–e).

My model predicts that Hsp70 chaperones with higher Hsp40-stimulated ATP hydrolysis rates can drive substrate folding to higher native fractions (Fig. 6e), at the cost of higher free energy expenditure (Fig. 6f). This result explains a previous experimental observation that a small molecule that enhances ATP hydrolysis by Hsp40-bound Hsp70 can induce higher yields of substrate folding^44^. Modulation of the ATP hydrolysis or the nucleotide exchange rates by small molecules may represent a therapeutic opportunity in the treatment of misfolding- or proteostasis-related diseases^45^.

## Discussion

Key to my model is the assumption that Hsp40 has different affinities for a substrate in different conformations, favoring the misfolding-prone conformation over the fold-competent conformation. This is supported by a number of experimental observations. Hsp40 binds to exposed hydrophobic sites using a Zn finger-like domain within its CTD^20^. It has been inferred from experiments that the binding of Hsp40 CTD to substrate is the strongest for unfolded peptides, weaker for partially unfolded proteins, and the weakest for native proteins, and that Hsp40 is able to distinguish the substrate conformations^22^. Hsp40 has been experimentally shown to bind to a number of denatured proteins, including denatured luciferase^20–22^, but it binds to few native proteins, notably to σ^32^, which adopts a loosely folded and highly flexible conformation^46^, and to RepE, the binding to which depends on the G/F-rich domain (outside CTD) of Hsp40 instead of the Zn finger-like domain^22^. These results suggest that Hsp40 preferentially binds to loose conformations with many exposed hydrophobic sites, which, together with the experimental observation that the fold-competent conformation is more compact with fewer exposed hydrophobic sites than the misfolding-prone conformation^4^, provides support for the assumption. Future experiments may directly test the assumption.

My model makes two distinct predictions that subject it to future experimental tests and possible falsification. First, it predicts that some thermodynamically unstable substrates depend on continuous Hsp70 assistance to maintain their native structures, and such a substrate in the steady state of Hsp70-mediated folding will gradually lose its native structure upon disruption of the chaperone system. Second, it predicts that an Hsp70 molecule bound to a substrate molecule will dissociate faster if the substrate is in the fold-competent conformation than if it is in the misfolding-prone conformation, and that this difference will disappear in the absence of Hsp40.

In support of the first prediction above, a recent experiment has demonstrated that luciferase at 37 °C can be kept active by the DnaK/DnaJ/GrpE chaperone system when there is sufficient ATP, but it rapidly loses its activity when ATP is depleted by the addition of apyrase^13^. The interpretation of this experiment, however, is complicated because apyrase also affects the luciferase activity assay. Here, based on my model, I propose an alternative experiment, in which Hsp70-mediated maintenance of luciferase activity is disrupted by inhibiting the simultaneous binding of Hsp40 to Hsp70 and to the substrate protein. For example, an isolated J-domain (e.g., DnaJ with its CTD deleted) can be used to compete against Hsp40 in binding to the Hsp70; alternatively, two D-peptides known to compete against substrate for binding to DnaJ, without binding to DnaK, can be used to inhibit DnaJ binding to the substrate^47^. When the J-domain or the D-peptide is added in excess to luciferase kept active by the DnaK/DnaJ/GrpE system, my model predicts that luciferase will lose its activity.

The second prediction of my model may be tested by kinetic experiments. Luciferase can be unfolded to different extent at different concentrations of the chemical denaturant guanidinium chloride (GdmCl): luciferase adopts a more compact unfolded structure with fewer exposed hydrophobic sites at lower concentrations of the denaturant than at higher concentrations of the denaturant^4^. My model predicts that, in the presence of Hsp40 and ATP, the dissociation rate of Hsp70 from luciferase denatured by low concentrations of GdmCl will be higher than from luciferase denatured at high concentrations of GdmCl, and that this difference in the dissociate rates will disappear in the absence of Hsp40.

Single molecule experiments^25,34^ may provide a more stringent test of the second prediction of my model, if one can monitor both the residence time of Hsp70 on a substrate molecule and the probability that the same substrate molecule subsequently folds into the native structure. My model predicts that in the presence of Hsp40 and ATP, the folding probability will be higher if the residence time is shorter, but this correlation will vanish in the absence of Hsp40. Such experiments may be feasible if, for instance, separate fluorescence signals to detect Hsp70-substrate binding and substrate folding become available.

## Methods

### Model of Hsp70-mediated protein folding

I denote Hsp70 as *C*, Hsp40 (J protein) as *J*, and the NEF as *E*. [*Y*] denotes the solution concentration of the molecular species *Y*. There are four types of reactions explicitly considered in my model (Fig. 1b):

1. Hsp70 binding to the substrate.
  1 $$S+C\cdot X\underset{{k}_{d,C\cdot X}}{\overset{{n}_{C}^{(S)}{k}_{a,C\cdot X}^{(0)}}{\rightleftharpoons }}S\cdot C\cdot X\,{\rm{for}}\,X=ATP,ADP$$
2. Conformational transitions of the substrate. An Hsp70-free substrate can adopt any of the four conformational states
  2 $$M\underset{[U]\cdot {k}_{U\to M}^{\dagger }([J])}{\overset{{k}_{M\to U}}{\rightleftharpoons }}U$$
  3 $$U\underset{{k}_{F\to U}}{\overset{{k}_{U\to F}^{\dagger }([J])}{\rightleftharpoons }}F$$
  4 $$F\underset{{k}_{N\to F}}{\overset{{k}_{F\to N}}{\rightleftharpoons }}N$$
  The chaperone-bound substrate can only be in and transition between the *U* and *F* states
  5 $$U\cdot C\cdot X\underset{{k}_{F\to U}}{\overset{{k}_{U\to F}^{\dagger }([J]){n}_{C}^{(F)}/{n}_{C}^{(U)}}{\rightleftharpoons }}F\cdot C\cdot X\,{\rm{for}}\,X=ATP,ADP$$
3. ATP hydrolysis.
  6 $$S\cdot C\cdot ATP\mathop{\to }\limits^{{k}_{h}^{(S)}([J])}S\cdot C\cdot ADP+Pi$$
4. Nucleotide exchange.

7 $$S\cdot C\cdot ADP+ATP\mathop{\to }\limits^{{k}_{e}([E])}S\cdot C\cdot ATP+ADP$$

The details of the kinetic rates of the above reactions are described below.

### Hsp70 binding to the substrate

The association rate constant of Hsp70 binding to a substrate in state S, ${k}_{a,C\cdot X}^{(S)}$, is determined by the number of accessible binding sites, ${n}_{C}^{(S)}$, in that conformation: ${k}_{a,C\cdot X}^{(S)}={n}_{C}^{(S)}{k}_{a,C\cdot X}^{(0)}$, where ${k}_{a,C\cdot X}^{(0)}$ is the association rate constant of the chaperone binding to a fully accessible binding site, and *X* = *ATP*, *ADP*. The dissociation rate constant of Hsp70 from the substrate, ${k}_{d,C\cdot X}$, does not depend on the substrate conformation, but depends on whether nucleotide *X* = *ATP* or *ADP* is bound. Experimentally, ${k}_{a,C\cdot ATP}^{(0)}\gg {k}_{a,C\cdot ADP}^{(0)}$ and ${k}_{d,C\cdot ATP}\gg {k}_{d,C\cdot ADP}$. I assume ${n}_{C}^{(U)} > {n}_{C}^{(F)}$.

### Conformational transitions of the substrate

The transition rates between conformations *S* and *S*′ are different between a chaperone-free substrate (${k}_{S\to S\text{'}})\,$and a chaperone-bound substrate (${k}_{S\cdot C\cdot X\to {S}^{\text{'}}\cdot C\cdot X}$) (Fig. 1b). The condition of thermodynamic cycle closure dictates that

8 $$\frac{{k}_{S\cdot C\cdot X\to {S}^{\text{'}}\cdot C\cdot X}}{{k}_{{S}^{\text{'}}\cdot C\cdot X\to S\cdot C\cdot X}}=\frac{{k}_{a,C\cdot X}^{({S}^{\text{'}})}}{{k}_{a,C\cdot X}^{(S)}}\frac{{k}_{S\to S\text{'}}}{{k}_{{S}^{\text{'}}\to S}}=\frac{{n}_{C}^{({S}^{\text{'}})}}{{n}_{C}^{(S)}}\frac{{k}_{S\to S\text{'}}}{{k}_{{S}^{\text{'}}\to S}}\,.$$

Because Hsp40 has different affinities for different substrate conformations, the transition rates between the conformations will depend on whether the substrate is bound to Hsp40. I treat the effects of Hsp40 on the reactions implicitly by making the affected rate constants dependent on the solution Hsp40 concentration [*J*] (see below).

For the transition $U\cdot C\cdot X\rightleftharpoons F\cdot C\cdot X$, I assume that the bound chaperone does not hinder the substrate to go from the *F* state to the *U* state, because a binding site available in the *F* state is most likely also available in the *U* state (based on the assumption ${n}_{C}^{(U)} > {n}_{C}^{(F)}$). Thus I take ${k}_{F\cdot C\cdot X\to U\cdot C\cdot X}={k}_{F\to U}$. It follows from thermodynamic cycle closure that the rate of the reverse transition—I use the superscript dagger to indicate that they are influenced by the presence of Hsp40—is

9 $${k}_{U\cdot C\cdot X\to F\cdot C\cdot X}^{\dagger }([J])={k}_{U\to F}^{\dagger }([J]){n}_{C}^{(F)}/{n}_{C}^{(U)}$$

I take the rate of (reversible) aggregation to be proportional to the substrate concentration:

10 $${k}_{U\to M}([U],[J])=[U]\cdot {k}_{U\to M}^{\dagger }([J])$$

The rates ${k}_{U\to S}^{\dagger }([J])$ (for *S* *=* *F*, *M*) depend on the affinities of Hsp40 for the substrate in different conformational states. For simplicity, I assume that Hsp40 only binds to the substrate in the *U* state, and consequently only the Hsp40-free substrate can change from conformation *U* to *F* or *M*. The corresponding transition rates are

11 $${k}_{U\to S}^{\dagger }([J])\approx (1-{p}_{J}([J]))\cdot {k}_{U\to S}\approx \frac{1}{1+[J]\cdot {K}_{A,J}^{(U)}}{k}_{U\to S},$$

where *k*_*U*→*S*_ is the rate of transition *U* → *S* (for *S* = *F*, *M*) for an Hsp40-free substrate, *p*_*J*_ is the probability that the substrate is Hsp40-bound, and ${K}_{A,J}^{(S)}$ is the binding constant of Hsp40 for the conformational state *S*.

### Hsp40-substrate binding

To keep my model simple, I do not explicitly consider the kinetics of binding and unbinding between Hsp40 and the substrate, and make the approximation that they are always at equilibrium. The key assumption of my model is that the fold-competent conformation *F* is much less accessible to Hsp40 than the misfolding-prone conformation *U*, i.e., ${K}_{A,J}^{(F)}\ll {K}_{A,J}^{(U)}$. To reduce the number of unknown parameters, I take ${K}_{A,J}^{(F)}\approx 0$, i.e., the binding of Hsp40 to the fold-competent conformation is negligible, as in our derivation of the transition rates above. I also neglect subtleties such as that the J domain may bind with different affinities to Hsp70 in the ATP- and ADP-states^48^. The above approximations may contribute to quantitative differences between the predictions of my model and the experimental observations, particularly in predicting how folding changes with Hsp40 concentrations. The binding and unbinding of Hsp40 to the substrate and to Hsp70 can be explicitly included in my model at the cost of greater complexity and additional fitting parameters, but my simplified treatment above is adequate for the key results in this work.

### ATP hydrolysis

The Hsp40-stimulated Hsp70 ATP hydrolysis rate, ${k}_{h}^{(J)}$, can be orders-of-magnitude higher than the unstimulated basal rate ${k}_{h}^{(0)}$. The ATP hydrolysis rate of Hsp70 bound to the substrate in the F state is simply ${k}_{h}^{(F)}([J])={k}_{h}^{(0)}$, following our approximation that no Hsp40 binds to the substrate in the *F* state. When Hsp70 is bound to the substrate in the *U* state, its average rate of ATP hydrolysis, given the solution Hsp40 concentration, [*J*], can be approximated by

12 $${k}_{h}^{(U)}([J])\approx {p}_{J}([J])(\frac{{K}_{h}}{1+{K}_{h}}{k}_{h}^{(J)}+\frac{1}{1+{K}_{h}}{k}_{h}^{(0)})+(1-{p}_{J}([J])){k}_{h}^{(0)},$$

where ${K}_{h}\equiv {k}_{a,J\cdot C}\cdot {[J]}_{\text{eff}}/({k}_{d,J\cdot C}+{k}_{h}^{(J)})$, with *k*_*a,J·C*_ and *k*_*d,J·C*_ being the association and dissociation rates of J domain binding to Hsp70, and [*J*]_eff_ being the effective concentration of a substrate-bound Hsp40 molecule around an Hsp70 molecule bound to the same substrate molecule. The first term on the right hand side is the steady state rate of catalysis, weighted by the probability *p*_*J*_ that an Hsp40 is bound to the substrate and thus present to catalyze the hydrolysis. Here I assume a high ATP concentration such that ATP binding to Hsp70 is fast compared to other steps in ATP hydrolysis.

### NEF-catalyzed nucleotide exchange

Because of the high concentration of ATP in cells and in the refolding experiments, I treat this reaction as irreversible. The reaction proceeds in three steps: (1) dissociation of ADP, (2) binding of ATP, and (3) conformational change of Hsp70 from the closed conformation in the ADP-state to the open conformation in the ATP-state. The conformational equilibrium between the open and closed conformations may be influenced by Hsp40 binding to Hsp70^49^, but this effect is not considered in my model for simplicity and lack of experimental parameters. In the absence of the nucleotide exchange factor, the rate limiting step in the reaction is the dissociation of ADP, with the rate constant *k*_*d,ADP*_, whereas when catalyzed by the NEF, the rate limiting step is the conformational change, with rate constant *k*_*C*_^29,50^. The overall rate of reaction at a given NEF concentration, [*E*], is then approximately

13 $${k}_{e}([E])=\frac{{K}_{e}}{1+{K}_{e}}{k}_{C}+\frac{1}{1+{K}_{e}}{k}_{d,ADP},$$

where ${K}_{e}\equiv {k}_{a,E\cdot C}\cdot [E]/({k}_{d,E\cdot C}+{k}_{C})$, with *k*_*a,E·C*_ and *k*_*d,E·C*_ being the association and dissociation rates of NEF binding to Hsp70. The temperature dependence of *k*_*C*_ for DnaK has been determined to satisfy the Arrhenius equation: ${k}_{C}(T)={k}_{C}^{(0)}\exp (\,\,-\,{E}_{a}/(RT))$, where *R* *=* 8.314 J/mol/K is the gas constant.

### Solving the kinetic equations

To simplify the calculations of refolding kinetics, I make the approximation that the solution concentrations of Hsp70, Hsp40, and NEF remain constant throughout the refolding process, which is true if they are in large excess of the substrate-bound chaperone, cochaperone, and NEF. Under this approximation, refolding kinetics is described by a set of linear ordinary differential equations, which are solved by the technique of eigenvalue decomposition of the rate matrix. This simplification allows quick and robust fitting of the folding kinetic parameters to the experimental refolding data. The steady state calculations do not use this approximation.

### Proof that without cochaperones Hsp70 cannot alter the population ratio between the native and the misfolded states

Consider a hypothetical, single-conformation substrate *s* and a reference reaction cycle of chaperone binding ($s+C\cdot X\rightleftharpoons s\cdot C\cdot X$ for *X* = *ATP*, *ADP*), ATP hydrolysis (*s· C · ATP* → *s · C · ADP* + *Pi*), and nucleotide exchange (*s · C · ADP* + *ATP* → *s · C · ATP* + *ADP*), where the ATP-bound and ADP-bound chaperones bind to *s* with the rate constants ${k}_{a,C\cdot ATP}^{(0)}$ and ${k}_{a,C\cdot ADP}^{(0)}$, respectively. Let *q*, *q*_*C·ATP*_ and *q*_*C·ADP*_ be the fractions of the hypothetical substrate that are chaperone-free (*s*), bound to an ATP-bound chaperone (*s · C · ATP*), and bound to an ADP-bound chaperone (*s · C · ADP*), respectively, at the steady state of this reaction cycle. For the real substrate in Hsp70-mediated folding, let *f*_*S*_ and *f*_*S·C·X*_ (*X* = *ATP*, *ADP*) be the fractions of the free and the *C · X*-bound substrates in conformation *S*, and let *f*_*S,eq*_ be the fraction of the substrate in conformation *S* at the folding equilibrium in the absence of the chaperones. It can be verified that

14 $${f}_{S}={Q}^{-1}{f}_{S,eq}q$$

15 $${f}_{S\cdot C\cdot X}={Q}^{-1}{f}_{S,eq}{n}_{C}^{(S)}{q}_{C\cdot X},\text{for}\,X=ATP,ADP$$

where

16 $$Q=\sum _{S}{f}_{S,eq}(q+{n}_{C}^{(S)}({q}_{C\cdot ATP}+{q}_{C\cdot ADP}))\,,$$

are the steady state solutions to the kinetic equations, given the condition of thermodynamic cycle closure in equation (8). Thus the ratio ${f}_{S}/{f}_{S\text{'}}={f}_{S,eq}/{f}_{{S}^{\text{'}},eq}$ is not altered by the chaperone, despite the free energy expenditure of ATP hydrolysis. This holds true for all numbers of intermediate states, so long as the ATP hydrolysis and the nucleotide exchange rates of the chaperone do not depend on the conformational state of the bound substrate.

## Data Availability

No datasets were generated or analyzed during the current study.
